# Supplementary material for: Modeling glioblastoma heterogeneity as a dynamic network of cell states
Source: Mol Syst Biol. 2021 Sep 16;17(9):e10105. doi: 10.15252/msb.202010105 (PMC8444284; doi:10.15252/msb.202010105)
Supplement: Supplementary file 6 — Source Data for Figure 5 [file MSB-17-e10105-s004.zip › Figure5A_sourcedata/GSEA_3017/nosigdb_stateC.GseaPreranked.1622795002785/FELDMAN_2020_S-G2.html]

Details for gene set FELDMAN\_2020\_S-G2[GSEA]

|  || Dataset | state13017 |
| Phenotype | NoPhenotypeAvailable |
| Upregulated in class | na\_pos |
| GeneSet | FELDMAN\_2020\_S-G2 |
| Enrichment Score (ES) | 0.4923918 |
| Normalized Enrichment Score (NES) | 3.953034 |
| Nominal p-value | 0.0 |
| FDR q-value | 0.0 |
| FWER p-Value | 0.0 |
Table: GSEA Results Summary

  

Fig 1: Enrichment plot: FELDMAN\_2020\_S-G2      
 Profile of the Running ES Score & Positions of GeneSet Members on the Rank Ordered List

  

| PROBE | GENE SYMBOL | GENE\_TITLE | RANK IN GENE LIST | RANK METRIC SCORE | RUNNING ES | CORE ENRICHMENT || 1 | SYNE2 |  |  | 0 | 0.839 | 0.0227 | Yes |
| 2 | ATAD2 |  |  | 7 | 0.649 | 0.0282 | Yes |
| 3 | HIST1H4C |  |  | 9 | 0.603 | 0.0425 | Yes |
| 4 | CENPU |  |  | 10 | 0.588 | 0.0584 | Yes |
| 5 | HIST1H1C |  |  | 11 | 0.583 | 0.0741 | Yes |
| 6 | HIST1H1D |  |  | 12 | 0.583 | 0.0899 | Yes |
| 7 | CLSPN |  |  | 17 | 0.561 | 0.0970 | Yes |
| 8 | RRM2 |  |  | 19 | 0.542 | 0.1097 | Yes |
| 9 | HMGB2 |  |  | 20 | 0.534 | 0.1241 | Yes |
| 10 | UBE2T |  |  | 31 | 0.475 | 0.1170 | Yes |
| 11 | HIST1H1B |  |  | 35 | 0.460 | 0.1234 | Yes |
| 12 | GMNN |  |  | 38 | 0.453 | 0.1317 | Yes |
| 13 | ORC6 |  |  | 39 | 0.450 | 0.1438 | Yes |
| 14 | ESCO2 |  |  | 43 | 0.440 | 0.1497 | Yes |
| 15 | RAD51AP1 |  |  | 46 | 0.435 | 0.1575 | Yes |
| 16 | PCNA |  |  | 48 | 0.435 | 0.1672 | Yes |
| 17 | TMPO |  |  | 49 | 0.432 | 0.1789 | Yes |
| 18 | HIST1H1E |  |  | 51 | 0.430 | 0.1885 | Yes |
| 19 | TOP2A |  |  | 52 | 0.426 | 0.2000 | Yes |
| 20 | SPC25 |  |  | 53 | 0.415 | 0.2112 | Yes |
| 21 | DHFR |  |  | 55 | 0.405 | 0.2202 | Yes |
| 22 | HIST1H2AH |  |  | 59 | 0.393 | 0.2248 | Yes |
| 23 | TYMS |  |  | 60 | 0.390 | 0.2353 | Yes |
| 24 | RRM1 |  |  | 62 | 0.386 | 0.2438 | Yes |
| 25 | SMC2 |  |  | 63 | 0.386 | 0.2542 | Yes |
| 26 | PSIP1 |  |  | 73 | 0.374 | 0.2463 | Yes |
| 27 | DEK |  |  | 74 | 0.373 | 0.2564 | Yes |
| 28 | NASP |  |  | 75 | 0.371 | 0.2664 | Yes |
| 29 | CENPH |  |  | 77 | 0.366 | 0.2743 | Yes |
| 30 | SMC4 |  |  | 78 | 0.365 | 0.2842 | Yes |
| 31 | SOX4 |  |  | 80 | 0.362 | 0.2920 | Yes |
| 32 | GINS2 |  |  | 83 | 0.360 | 0.2977 | Yes |
| 33 | STMN1 |  |  | 85 | 0.357 | 0.3053 | Yes |
| 34 | DUT |  |  | 87 | 0.356 | 0.3130 | Yes |
| 35 | MIS18BP1 |  |  | 89 | 0.355 | 0.3205 | Yes |
| 36 | CARHSP1 |  |  | 90 | 0.353 | 0.3301 | Yes |
| 37 | MCM7 |  |  | 91 | 0.352 | 0.3396 | Yes |
| 38 | USP1 |  |  | 92 | 0.352 | 0.3491 | Yes |
| 39 | ATAD5 |  |  | 97 | 0.345 | 0.3504 | Yes |
| 40 | FEN1 |  |  | 98 | 0.342 | 0.3597 | Yes |
| 41 | NUSAP1 |  |  | 100 | 0.340 | 0.3668 | Yes |
| 42 | MCM3 |  |  | 114 | 0.324 | 0.3497 | Yes |
| 43 | H2AFX |  |  | 115 | 0.322 | 0.3584 | Yes |
| 44 | HIRIP3 |  |  | 116 | 0.321 | 0.3670 | Yes |
| 45 | PSMC3IP |  |  | 118 | 0.319 | 0.3737 | Yes |
| 46 | CCDC34 |  |  | 120 | 0.318 | 0.3803 | Yes |
| 47 | HAT1 |  |  | 121 | 0.318 | 0.3889 | Yes |
| 48 | SMC1A |  |  | 125 | 0.312 | 0.3913 | Yes |
| 49 | HMGN2 |  |  | 126 | 0.312 | 0.3997 | Yes |
| 50 | DNMT1 |  |  | 127 | 0.311 | 0.4081 | Yes |
| 51 | FBXO5 |  |  | 130 | 0.308 | 0.4124 | Yes |
| 52 | NCAPG |  |  | 137 | 0.301 | 0.4086 | Yes |
| 53 | RFC4 |  |  | 138 | 0.300 | 0.4167 | Yes |
| 54 | CENPK |  |  | 139 | 0.299 | 0.4248 | Yes |
| 55 | LMNB1 |  |  | 142 | 0.293 | 0.4287 | Yes |
| 56 | ASF1B |  |  | 143 | 0.291 | 0.4366 | Yes |
| 57 | HNRNPAB |  |  | 144 | 0.290 | 0.4444 | Yes |
| 58 | SIVA1 |  |  | 151 | 0.286 | 0.4402 | Yes |
| 59 | SUPT16H |  |  | 153 | 0.286 | 0.4459 | Yes |
| 60 | RNASEH2B |  |  | 158 | 0.282 | 0.4455 | Yes |
| 61 | CEP78 |  |  | 159 | 0.281 | 0.4531 | Yes |
| 62 | HIST2H2AC |  |  | 161 | 0.281 | 0.4587 | Yes |
| 63 | PARP1 |  |  | 163 | 0.280 | 0.4643 | Yes |
| 64 | MYBL2 |  |  | 164 | 0.278 | 0.4718 | Yes |
| 65 | VRK1 |  |  | 169 | 0.275 | 0.4713 | Yes |
| 66 | HELLS |  |  | 171 | 0.271 | 0.4766 | Yes |
| 67 | C19orf48 |  |  | 173 | 0.271 | 0.4819 | Yes |
| 68 | MAD2L1 |  |  | 174 | 0.270 | 0.4892 | Yes |
| 69 | RBBP8 |  |  | 177 | 0.267 | 0.4924 | Yes |
| 70 | CDK1 |  |  | 191 | 0.255 | 0.4734 | No |
| 71 | UPP1 |  |  | 204 | -0.253 | 0.4563 | No |
| 72 | SAT1 |  |  | 241 | -0.262 | 0.3917 | No |
| 73 | BAMBI |  |  | 289 | -0.273 | 0.3054 | No |
| 74 | PLAU |  |  | 294 | -0.274 | 0.3049 | No |
| 75 | PHLDA1 |  |  | 295 | -0.274 | 0.3123 | No |
| 76 | TMEM158 |  |  | 315 | -0.284 | 0.2821 | No |
| 77 | IGFBP5 |  |  | 384 | -0.311 | 0.1550 | No |
| 78 | PLP1 |  |  | 386 | -0.312 | 0.1615 | No |
| 79 | RPL22L1 |  |  | 404 | -0.322 | 0.1363 | No |
| 80 | KLF6 |  |  | 429 | -0.339 | 0.0976 | No |
| 81 | RAB13 |  |  | 482 | -0.388 | 0.0045 | No |
| 82 | RNF19A |  |  | 485 | -0.388 | 0.0110 | No |
| 83 | ARL6IP5 |  |  | 490 | -0.396 | 0.0137 | No |
| 84 | FABP7 |  |  | 495 | -0.411 | 0.0168 | No |
| 85 | SCRG1 |  |  | 508 | -0.434 | 0.0047 | No |
| 86 | LYPD1 |  |  | 521 | -0.454 | -0.0070 | No |
| 87 | IFITM3 |  |  | 533 | -0.477 | -0.0160 | No |
| 88 | IGFBP3 |  |  | 538 | -0.493 | -0.0107 | No |
| 89 | TTYH1 |  |  | 540 | -0.496 | 0.0007 | No |
| 90 | TNFRSF12A |  |  | 550 | -0.519 | -0.0032 | No |
| 91 | PLAT |  |  | 556 | -0.538 | 0.0013 | No |
| 92 | S100B |  |  | 561 | -0.561 | 0.0085 | No |
| 93 | SPARC |  |  | 567 | -0.575 | 0.0141 | No |
| 94 | CLU |  |  | 581 | -0.690 | 0.0068 | No |
| 95 | CTGF |  |  | 588 | -0.782 | 0.0159 | No |
Table: GSEA details [plain text format]

  

Fig 2: FELDMAN\_2020\_S-G2: Random ES distribution      
 Gene set null distribution of ES for **FELDMAN\_2020\_S-G2**
